# Ocular Co-Delivery of Timolol and Brimonidine from a Self-Assembling Peptide Hydrogel for the Treatment of Glaucoma: In Vitro and Ex Vivo Evaluation

**DOI:** 10.3390/ph13060126

**Published:** 2020-06-21

**Authors:** Elissavet Taka, Christina Karavasili, Nikolaos Bouropoulos, Thomas Moschakis, Dimitrios D. Andreadis, Constantinos K. Zacharis, Dimitrios G. Fatouros

**Affiliations:** 1 Department of Pharmaceutical Technology, School of Pharmacy, Aristotle University of Thessaloniki, GR-54124 Thessaloniki, Greece; takaelis@pharm.auth.gr (E.T.); dfatouro@pharm.auth.gr (D.G.F.); 2 Foundation for Research and Technology Hellas, Institute of Chemical Engineering and High Temperature Chemical Processes, GR-26504 Patras, Greece; nbouro@upatras.gr; 3 Department of Materials Science, University of Patras, GR-26504 Patras, Greece; 4 Department of Food Science and Technology, School of Agriculture, Aristotle University, GR-54124 Thessaloniki, Greece; tmoschak@agro.auth.gr; 5 Department of Oral Medicine/Pathology, School of Dentistry, Aristotle University of Thessaloniki, GR-54124 Thessaloniki, Greece; dandrea@dent.auth.gr; 6 Laboratory of Pharmaceutical Analysis, Department of Pharmaceutical Technology, School of Pharmacy, Aristotle University of Thessaloniki, GR-54124 Thessaloniki, Greece; czacharis@pharm.auth.gr

**Keywords:** self-assembling peptide hydrogel, glaucoma, timolol maleate/brimonidine tartrate combination, ex vivo ocular permeability

## Abstract

Effective pharmacotherapy during glaucoma treatment depends on interventions that reduce intraocular pressure (IOP) and retain the IOP lowering effect for sufficient time so as to reduce dosing frequency and enhance patient adherence. Combination anti-glaucoma therapy and dosage forms that increase precorneal residence time could therefore constitute a promising therapeutic intervention. The in-situ gel forming self-assembling peptide ac-(RADA)_4_-CONH_2_ was evaluated as carrier for the ocular co-delivery of timolol maleate (TM) and brimonidine tartrate (BR). The hydrogel’s microstructure and mechanical properties were assessed with atomic force microscopy and rheology, respectively. Drug diffusion from the hydrogel was evaluated in vitro in simulated tear fluid and ex vivo across porcine corneas and its effect on the treated corneas was assessed through physicochemical characterization and histological analysis. Results indicated that TM and BR co-delivery affected hydrogel’s microstructure resulting in shorter nanofibers and a less rigid hydrogel matrix. Rapid and complete release of both drugs was achieved within 8 h, while a 2.8-fold and 5.4-fold higher corneal permeability was achieved for TM and BR, respectively. No significant alterations were induced in the structural integrity of the corneas treated with the hydrogel formulation, suggesting that self-assembling peptide hydrogels might serve as promising systems for combination anti-glaucoma therapy.

## 1. Introduction

Local instillation is the most preferred non-invasive route of administration for the treatment of eye diseases, such as glaucoma [1]. Conventional dosage forms, such as eye drops, account for the majority (ca. 90%) of the commercially available ophthalmic preparations, due to ease of self-administration which significantly enhances patient compliance to treatment. However, the presence of numerous anatomical barriers (such as choroidal and retinal epithelium) and physiological limitations (such as the tear cycle and lymphatic flow to the conjunctiva) constitute significant challenges to ophthalmic drug absorption, resulting in low ocular bioavailability after topical administration. As a result, no more than 5% of the topically instilled doses penetrate to deeper eye tissues [2]. 

In recent years, research on ophthalmic drug administration has oriented towards the development of new dosage forms and devices for drug administration, aiming to overcome these challenges and achieve higher drug levels in intraocular tissues [3]. Among those, in-situ gelling systems that are able to transit from sol to gel state upon physiological stimuli have shown great capacity in prolonging the corneal residence time, and therefore increasing drug absorption and ocular bioavailability and decreasing dosing frequency [4]. Hydrogels made up of self-assembling amphiphilic peptides have gained popularity in biomedical applications, such as drug delivery systems, due to their unique properties [5]. They comprise of repetitive patterns of ionic hydrophilic and hydrophobic natural amino acids rendering them biocompatible and biodegradable. Upon interaction with electrolyte solutions (either by adding a salt solution or by injecting the material in vivo), these peptides self-assemble through the formation of non-covalent transverse bonds between the fibers to form three-dimensional networks with high water content of up to 97–99.9% w/v [6,7]. Among the well-studied self-assembling peptides, RADA16-I was synthesized in resemblance to a 16-residue peptide (EAK16) segment of yeast origin (zuotin), that was found to form macroscopic membranes of fibrous architecture [8]. RADA16-I consists of the amino acid sequence CH_3_CO-RADARADARADARADA-CONH_2_ (R: arginine, A: alanine, D: aspartic acid) and includes tandem repeats of hydrophobic and charged amino acid residues that favor the adoption of b-sheet configurations. During self-assembly, b-sheets are arranged in a highly oriented nanoscale structure consisting of β-sheet bilayers stacked together through hydrophobic interactions developed by lateral alanine chains. The peptide solution RADA16-I forms hydrogels of high-water content [up to 99.5% (w/w)], with a pore diameter between 5 and 200 nm, allowing entrapment and gradual release of small molecules [9,10,11,12] and macromolecules [13,14,15].

Glaucoma is one of the leading causes of vision loss after cataract with a high prevalence mainly among elderly people. It is an irreversible optic neuropathy that involves progressive degeneration of the retinal ganglion cells resulting in structural changes in the optic nerve and is commonly associated with increased intraocular pressure (IOP) [16]. Due to the complex nature of glaucoma, a single active compound may be insufficient to effectively reduce IOP. Hence, combination of more than one glaucoma medication could prove advantageous not only in terms of IOP reduction, but also as a means to reduce dosing frequency and therefore enhance patient compliance and treatment adherence [17]. Brimonidine tartrate (BR), an α-2-adrenergic receptor agonist, and timolol maleate (TM), a β-1 and β-2 non-selective adrenergic receptor inhibitor, are two of the active compounds widely used to control IOP. Several studies have probed the synergistic efficacy of this combination in reducing IOP compared to the individual therapeutic effects of the two actives [18] and better therapeutic efficacy compared to various other combinations, such as dorzolamide / timolol [19]. Combigan^®^ is the only marketed ophthalmic combination of brimonidine tartrate and timolol maleate in solution form. Research on alternative dosage forms of brimonidine tartrate and timolol maleate combination is limited to the development of a polycaprolactone thin-film intraocular implant with significant IOP-lowering efficacy over 3 months in vivo [20], a stimulus-sensitive hydrogel of poly acrylic acid showing sustained IOP-lowering effects up to 12 h [21] and a polyamidoamine dendrimer hydrogel/poly(lactic-co-glycolic acid) nanoparticle platform achieving IOP-lowering effects for four days following single topical administration [22]. 

In the current study, the in-situ gel forming self-assembling peptide ac-(RADA)_4_-CONH_2_ was evaluated as a carrier of the anti-glaucoma drug combination of brimonidine tartrate and timolol maleate as a viable alternative to conventional eye-drops. 

## 2. Results and Discussion

### 2.1. Rheological Studies

**Figure 1:**
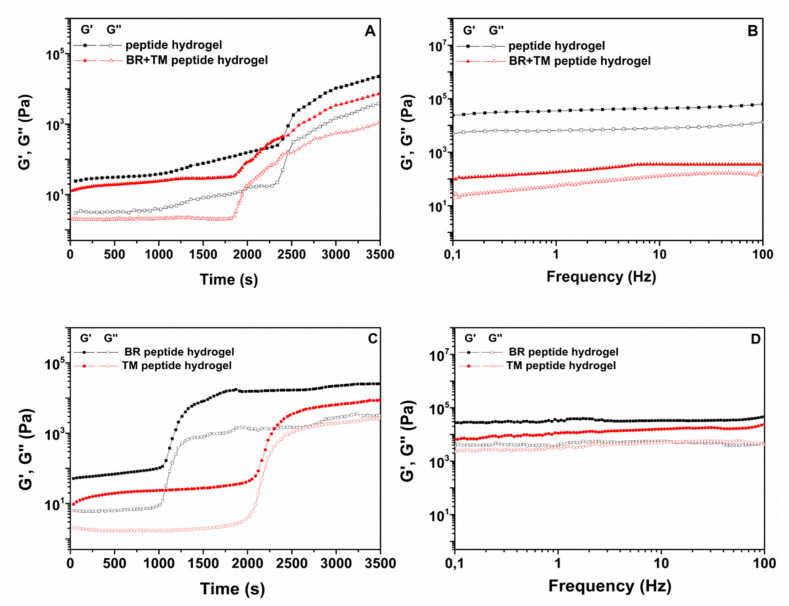
(**A**), (**C**). Oscillatory time sweep (0.5% strain, 1 Hz, 37 °C) and (**B**), **(D**). frequency sweep experiments (0.5% strain, 0.1–100 Hz, 37 °C) of the ac-(RADA)_4_-CONH_2_ peptide hydrogel and the single (BR, TM) and dual (TM + BR) drug loaded peptide hydrogel.

The gelation kinetics and viscoelastic properties of the ac-(RADA)_4_-CONH_2_ peptide hydrogel prior and post drug loading were evaluated with oscillatory time sweep (Figure 1A) and frequency sweep experiments (Figure 1B). In order to further elucidate the effect of each drug compound on the rheological properties of the peptide hydrogel, the same studies were conducted on the single drug loaded peptide hydrogel (TM peptide hydrogel, BR peptide hydrogel). The storage modulus (G’), which provides a measure of the materials elasticity, and the loss modulus (G’’), which is a measure of the deformation energy that is dissipated (viscous behavior), were plotted logarithmically against time and frequency. G’ max and tan δ values at t = 3500 s and the gelation onset values are presented in Table 1. The kinetic motif of the plain peptide hydrogel indicates that the onset of gelation, associated with an abrupt increase in G’, occurred at 39 min from the addition of the phosphate-buffered solution (PBS) solution, whereas for the drug loaded peptide hydrogel an earlier onset of gelation was observed at 30 min. As is evident in Figure 1C, BR induced an earlier onset of ac-(RADA)_4_-CONH_2_ gelation at 18 min, in contrast to TM for which gelation onset was observed at 35 min, indicating that the effect of BR was more pronounced on the gelation kinetics of the BR + TM peptide hydrogel. It is possible that BR as a polyaromatic hydrocarbon favors the hydrophobic interactions between the peptide nanofibers, resulting in a stiffer but lower elasticity (G’_max_:25.3, tan δ:0.13) hydrogel compared to the blank peptide hydrogel, which showed the most dominant elastic behavior (G’_max_:22.6, tan δ:0.05). For both plain and drug loaded peptide hydrogel, G’ values were an order of magnitude higher than G’’ throughout the experiment, indicative of a gel structure. Immediately after the gel curing experiments, the viscoelastic properties of the peptide hydrogel prior and post drug loading were monitored over the frequency range of 0.1 Hz to 100 Hz at 0.5% strain. Results revealed a dominant elastic behavior for both the plain and the drug loaded peptide hydrogel with G’ and G’’ being nearly independent of frequency (Figure 1B). However, the incorporation of TM and BR resulted in a decrease in hydrogel rigidity, as suggested by the higher G’ values obtained for the plain peptide hydrogel. This could be attributed to the possible stereochemical inhibition in the interaction between the peptide nanofibers caused by the simultaneous incorporation of the two active compounds within the peptide hydrogel, as also previously reported [10]. Evaluating the effect of individual drug on the strength of the formed hydrogel (Figure 1D), it is evident that BR has a marginal effect on hydrogel’s rigidity, as compared to TM. It is however, the combination of both in the peptide hydrogel that results in the decrease in hydrogel’s stiffness.

### 2.2. Atomic Force Microscopy (AFM) Characterization

**Figure 2:**
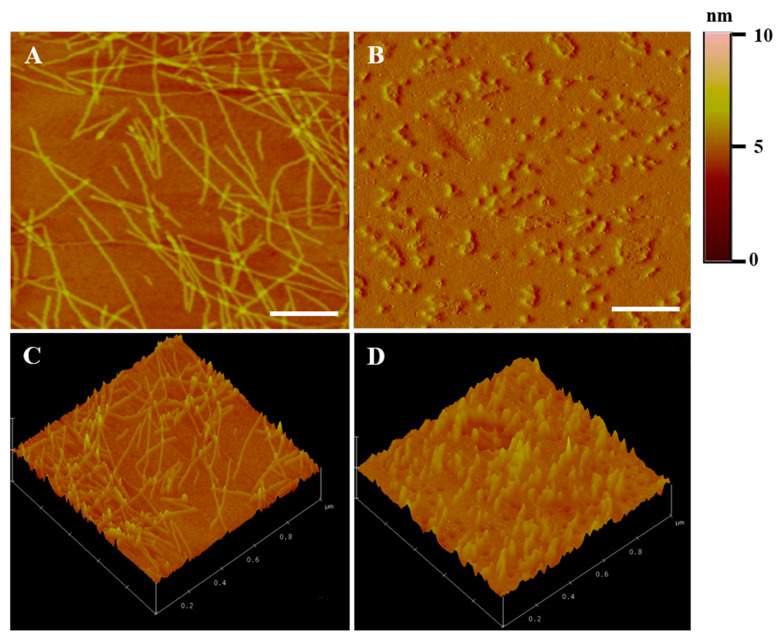
(**A**,**B**) 2D and (**C**,**D**) 3D AFM images of the ac-(RADA)_4_-CONH_2_ peptide hydrogel (**A**–**C**) prior and (**B**–**D**) post drug loading. Scale bar: 200 nm.

The nanofiber morphology of the ac-(RADA)_4_-CONH_2_ prior and post drug loading was visualized with AFM analysis. As shown in Figure 2A, ac-(RADA)_4_-CONH_2_ self-assembles into long nanofibers [23]. After drug loading (Figure 2B), peptide nanofibers that were shorter in length compared to those of the control sample were observed, which probably justifies the lower rigidity of the formed hydrogel, as already verified during rheological analysis. It is possible that the dual drug loading might have affected the self-assembly process creating steric hindrance in the peptide nanofiber interaction. The 3D plots of the peptide nanofibers prior and post drug loading are shown in Figure 2C,D, respectively.

### 2.3. In Vitro Release Study of TM and BR from the Self-Assembling Peptide ac-(RADA)_4_-CONH_2_ in Simulated Tear Fluid (STF)

**Figure 3:**
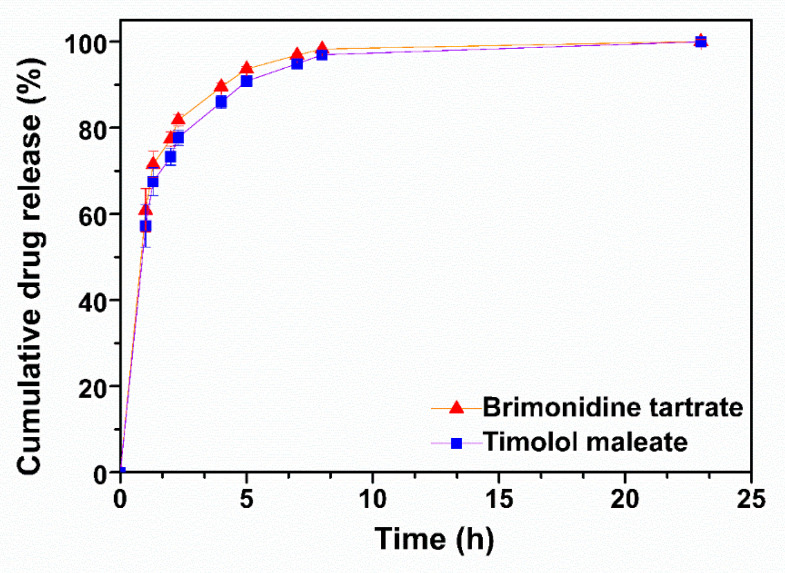
In vitro release profiles of brimonidine tartrate and timolol maleate from the ac-(RADA)_4_-CONH_2_ peptide hydrogel in STF pH 7.4 at 37 °C (*n* = 4).

The in vitro release profiles of TM and BR from the peptide hydrogel were recorded in STF pH 7.4 at 37 °C. Both drugs demonstrated similar release profiles (Figure 3) with a burst release of approximately 60% occurring within the first hour, achieving total release over a period of 8 h, in accordance to previous studies [11,21]. TM release from the peptide hydrogel was slightly slower, possibly due to the minor difference in its lipophilicity compared to BR (logP TM = 1.83; logP BR = 1.37), however this difference was not statistically significant.

### 2.4. Ex Vivo Corneal Permeability Studies

**Figure 4:**
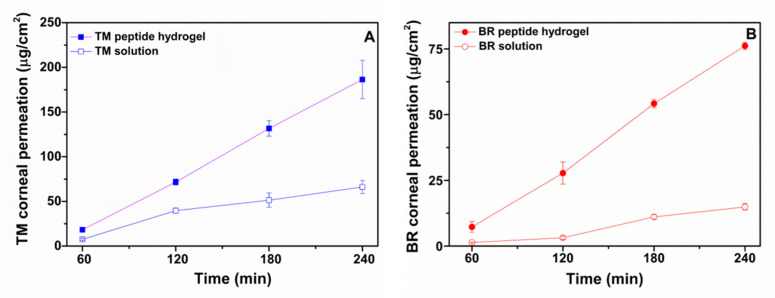
Ex vivo permeability profiles of (**A**). timolol maleate in ac-(RADA)_4_-CONH_2_ peptide hydrogel and in PBS solution and of (**B**). brimonidine tartrate in ac-(RADA)_4_-CONH_2_ peptide hydrogel and in PBS solution across porcine cornea (*n* ≥ 4).

The ex vivo permeability profiles of TM and BR solution and peptide hydrogel formulations across porcine corneas and the respective steady state flux (J_ss_) and apparent permeability values (P_app_) are shown in Figure 4 and Table 2, respectively. TM demonstrated a significantly higher permeability across corneal tissue when loaded within the peptide hydrogel (Figure 4A), with the amount of drug permeated at 4 h being 2.8 times higher for the peptide hydrogel formulation (186 ± 21 μg/cm^2^) than for the solution form (66 ± 7 μg/cm^2^). A similar trend was also observed for BR which showed a 5.4-fold higher amount of drug permeation at 4h from the peptide hydrogel (76 ± 1.3 μg/cm^2^) than from the solution (14.9 ± 1.2 μg/cm^2^). The P_app_ of TM in the peptide hydrogel and in solution were calculated to be 2.3 × 10^−6^ ± 0.3 cm/s and 0.75 × 10^−6^ ± 0.053 cm/s, respectively. Both values were found to be similar compared to the respective values reported in the literature for TM in the peptide hydrogel (1.91 × 10^−6^ ± 0.11 cm/s) [11] and in solution form (0.9 × 10^−6^ cm/s) [24]. The P_app_ of BR in the peptide hydrogel was calculated to be 3.3 × 10^−6^ ± 0.3 cm/s, which was almost five times higher than the P_app_ value calculated for BR in solution (0.63 × 10^−6^ ± 0.055 cm/s). It is possible that the short peptide nanofibers might act as permeation enhancers by physically perturbing the corneal epithelium to promote drug transport, therefore justifying the significantly higher permeability observed for both drugs from the peptide hydrogel formulation compared to the solution form.

### 2.5. Corneal Tissue Characterization Studies

#### 2.5.1. Fourier Transform Infrared Spectroscopy (FTIR) and Differential Scanning Calorimetry Analysis (DSC)

**Figure 5:**
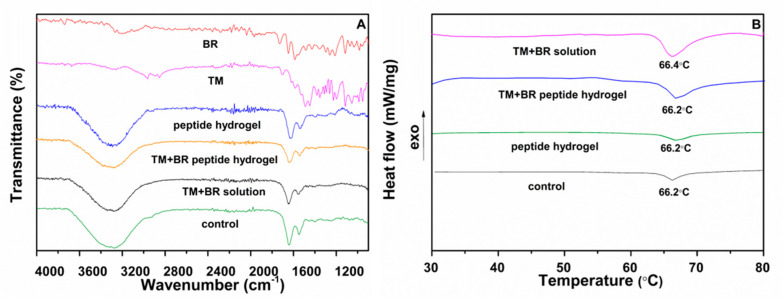
(**A**) FTIR spectra of pure brimonidine tartrate, pure timolol maleate and of the control cornea and the corneas treated with the peptide hydrogel, the drug solution and the drug loaded peptide hydrogel. (**B**) Thermograms of the control cornea and the corneas treated with the peptide hydrogel, the drug solution and the drug loaded peptide hydrogel.

FTIR is a valuable tool for detecting structural changes in collagen fibers, which together with water are the main components of the stroma layer in cornea [25]. Areas in the FTIR spectrum corresponding to the peaks of amides I, II, and III facilitate the detection of changes in the secondary structure of proteins, which are associated with tissue damage [25]. The FTIR spectra of the control and treated corneas with the TM and BR formulations are showed in Figure 5A. The peak at 1643 cm^−1^ is attributed to the vibration stretching of amide I (ν C = O) while the peak of amide II is located at 1548 cm^−1^ and is due to the vibrational bending of the N-H bond. The peaks of the amide I and II in the spectra of the corneal tissues treated with the TM and BR formulations were not differentiated from the corresponding control tissue and the tissue treated with the peptide hydrogel, possibly indicating the retainment of the structural integrity of the collagen fibers during the ex vivo permeability study. The characteristic absorption peaks of timolol maleate (2980 cm^−1^ C-H stretching) and brimonidine tartrate (1595 cm^−1^; N-H bending, 1649 cm^−1^; N = C and C = C bending) did not appear in the FTIR spectra of the treated corneas. 

DSC analysis was performed for the detection of possible changes in the structural integrity of the tissues after their interaction with the drug loaded peptide hydrogel during the ex vivo permeability study. The corneal tissue presents a phase transition due to structural changes in the network of collagen fibers in the stroma (unfolding of the structure of the triple helix of the protein) [26]. The thermal decomposition of collagen fibers depends on factors such as the water content of the tissue, the pH of the surrounding medium, the presence of salts, and the degree of interconnection of the fibers. High transition temperatures are associated with increased collagen fiber stability due to possible tissue dehydration [27]. In addition, the development of interactions between the cornea and the evaluated drug formulations may be reflected in the tissue thermograms as changes in the transition point or peak enthalpy [28]. The transition temperature of the intact porcine cornea is located at ca. 66 °C [29]. The thermograms of the corneal tissues treated with the drug loaded peptide hydrogel (Figure 5B) demonstrated a transition peak at 66.2 °C, with negligible differences in peak enthalpy and shape relative to the control sample and the cornea treated with the peptide hydrogel, while the transition temperature of the cornea treated with the TM + BR solution showed a minor shift of the peak at 66.4 °C. In conclusion, the thermal analysis of the tissues confirmed the maintenance of the structural integrity of the corneas treated with the drug loaded peptide hydrogel. 

#### 2.5.2. Hydration Content

The corneal hydration content provides a means to assess the integrity of the epithelial and endothelial layers. According to the literature, the normal humidity levels of intact tissues range from 73% to 80% [30] with the moisture content increasing above normal limits in cases of tissue integrity loss [31]. In the present study, the corneal hydration content was determined after the completion of the permeability study and after drying the tissues to a constant weight (Table 3) and was found to be in accordance with hydration levels for structurally intact tissues.

### 2.6. Histological Studies

**Figure 6:**
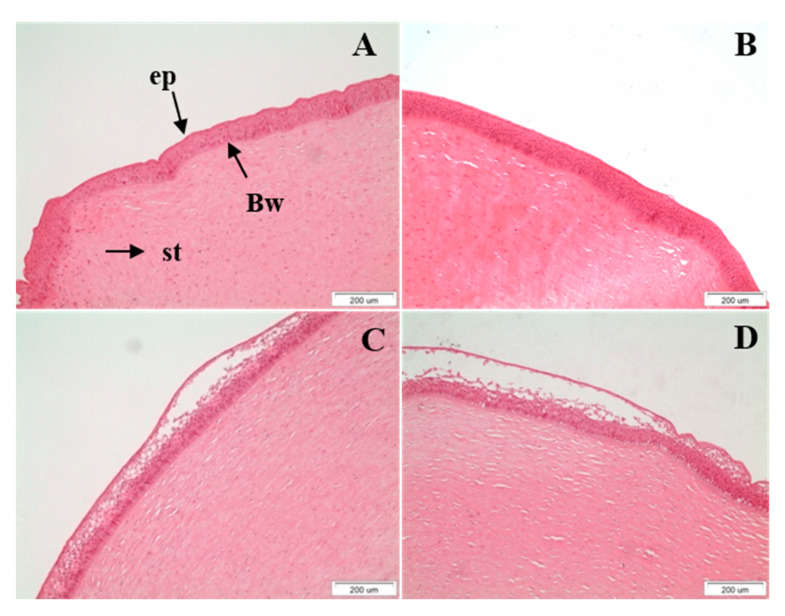
Histological sections of the (**A**). control (untreated) cornea, (**B**). cornea treated with the peptide hydrogel, (**C**). cornea treated with TM + BR solution and (**D**). cornea treated with TM + BR peptide hydrogel after the completion of the ex vivo permeability studies (ep: epithelium; Bw: Bowman’s layer; st: stroma). Scale bar: 200 μm.

The structural integrity of the treated corneas was additionally evaluated with histological analysis after the completion of the ex vivo permeability studies. Histological images are shown in Figure 6. The control cornea (Figure 6A) and the cornea treated with the peptide hydrogel (Figure 6B) demonstrated the morphology of intact tissue comprising of distinct layers of the epithelium, the Bowman layer, and the stroma without showing any sign of alteration in their structural integrity. A moderate detachment of the apical layer of the epithelium was observed for the corneal tissue interacting with the drugs solution (Figure 6B). This effect was relatively more pronounced for the cornea treated with the TM + BR peptide hydrogel formulation, for which minor spongiosis was also observed in the stroma layer. Clinical studies in healthy subjects have previously shown that the maleate salt of timolol can potentially induce corneal irritation after topical application [32]. This might justify the histological observations of the current study for the corneas treated with the drug loaded formulations. At the same time the higher TM permeability observed for the peptide hydrogel may have resulted in higher drug concentration on the corneal epithelium during the ex vivo study, further supporting the comparatively higher degree of irritation induced by the hydrogel formulation. However, the static nature of the Franz cell assembly from which the normal functions of the eye, such as movement of the eyelids and nasolacrimal fluid drainage, are absent and may have resulted in an overestimation of the irritant action of the drug loaded peptide hydrogel. This hypothesis was further supported in a previous study, in which the in vivo histological findings showed no structural changes in the corneal integrity after treatment with the TM loaded formulations in contradiction with the mild irritation effects observed in the ex vivo histological findings [11].

## 3. Materials and Methods 

### 3.1. Materials

Timolol maleate (TM, purity > 98%) and brimonidine tartrate (BR, purity > 98%) were purchased from Sigma-Aldrich (Germany). Ac-(RADA)_4_-CONH_2_ peptide solution (1% w/v) was obtained from 3D-Matrix Inc. (Japan). All other reagents were of analytical grade.

### 3.2. Preparation of the Drug Loaded Peptide Hydrogel

Ac-(RADA)_4_-CONH_2_ is a 16 amino-acid peptide solution consisting of 99% water content (10 mg/mL peptide content). Brimonidine tartrate and timolol maleate were dissolved in the peptide solution at the same concentrations used in the commercial ophthalmic solution Combigan™ (0.2% w/v for BR and 0.5% w/v for TM). Gelation was initiated upon the addition of phosphate buffered saline pH 7.4 (1X, 162 mM) to a final peptide concentration of 0.9% w/v.

### 3.3. Rheological Studies

Rheological measurements were conducted on a Physica MCR 300 rheometer (Physica Messtechnic GmbH, Stuttgart, Germany) equipped with a cone plate (diameter 25 mm, cone angle 1°) at a gap of 0.05 mm. Temperature control (37 ± 0.1 °C) was achieved using a Paar Physica circulating bath and a controlled Peltier system (TEZ 150P/MCR). A quantity of 600 μL of the blank and the drug loaded peptide solution was loaded on the rheometer plate and hydrated with PBS pH 7.4 to a final peptide concentration of 0.9% w/v in order to initiate the gelation process. Oscillatory time sweeps were then immediately carried out at a strain of 0.5% and a frequency of 1Hz, followed by frequency sweeps at a constant strain of 0.5% in the frequency region of 0.1–100 Hz. 

### 3.4. Atomic Force Microscopy (AFM) Studies

The effect of drug loading on the nanofiber morphology was visualized with AFM analysis. The plain and the drug loaded ac-(RADA)_4_-CONH_2_ peptide solution were diluted with Milli-Q water to a final concentration 0.01% w/v. Ten μL of the peptide solution were then deposited on a freshly cleaved mica and after 30 s rinsed with 300 μL Milli-Q water. AFM observations of the dried micas were conducted with a multimode scanning probe microscope (Veeco) with a Nanoscope IIIa controller. The spring constant of the cantilever was 10 N/m and height images were acquired with a 1 Hz scanning rate. 3D surface images were derived from the corresponding height images using the Nanoscope ver. 12.5 software.

### 3.5. In Vitro Release of TM and BR from the Self-Assembling Peptide Hydrogel in STF

In vitro release of TM and BR from the ac-(RADA)_4_-CONH_2_ peptide hydrogel was performed in STF pH 7.4 (NaCl, 6.8 g/L; NaHCO_3_, 2.2 g/L; CaCl_2_· 2H_2_O, 0.08 g/L; KCl, 1.4 g/L). Briefly, brimonidine tartrate and timolol maleate were dissolved in 45 μL of the peptide solution in an Eppendorf tube so as to obtain a final drug concentration of 0.2% w/v brimonidine tartrate and 0.5% w/v timolol maleate (final hydrogel volume: 50 μL). Gelation was initiated after the addition of 5 μL PBS pH 7.4 (NaCl, 8 g/L; Na_2_HPO_4_, 1.44 g/L; KCl, 0.2 g/L; KH_2_PO_4_, 0.24 g/L) resulting to a final peptide concentration of 0.9% w/v. After 60 min, 1 mL STF preconditioned at 37 °C was added slowly on top of the hydrogel and the tubes were placed in a water bath at 37 °C. At predetermined time points 800 μL aliquots were collected from the supernatant solutions and replaced with equal volume of STF buffer (37 °C). The withdrawn samples were analyzed with HPLC. The experiment was repeated four times.

### 3.6. Ex Vivo Corneal Permeability Studies

Ex vivo permeability studies were performed in Franz cells (diffusion area: 0.19 cm^2^, acceptor volume: 3 mL) modified for corneal delivery (Permegear, Hellertown, PA, USA). Corneas were isolated from freshly excised porcine eyes obtained from a local slaughterhouse. Macroscopically intact tissues were placed between the donor and the acceptor compartments. Glutathione bicarbonate Ringer’s solution (pH 7.4) was added in the acceptor chamber and maintained at 37 ˚C under mild magnetic stirring. For the preparation of the GBR solution equal volumes of solution A and B were mixed: Solution A consisted of NaCl (12.4 g/L), KCl (0.716 g/L), NaHCO_3_ (4.908 g/L) and NaH_2_PO_4_·H_2_O (0.206 g/L) and solution B consisted of MgCl_2_·6H_2_O (0.318 g/L), CaCl_2_·2H_2_O (0.23 g/L), glucose (1.8 g/L) and oxidized glutathione (0.184 g/L). Brimonidine tartrate and timolol maleate were dissolved in 90 μL of ac-(RADA)_4_-CONH_2_ peptide solution (to obtain a final drug concentration of 0.2% w/v brimonidine and 0.5% timolol in a final hydrogel volume of 100 μL), placed in the donor compartment and hydrated with 10 μL of PBS solution (0.9% w/v final peptide concentration) to initiate gelation. Permeability experiments were also performed for the solution of the drugs’ combination. Brimonidine tartrate and timolol maleate were dissolved in 100 μL PBS pH 7.4 to a final drug concentration of 0.2% w/v brimonidine tartrate and 0.5% timolol maleate. Samples (400 μL) were collected from the acceptor and replaced with equal volume of preconditioned at 37 °C GBR solution. All the experiments were performed in quadruplicates. Untreated corneas were used as control. Corneas were also treated with the peptide hydrogel. Drug content in the samples was quantified with HPLC analysis.

### 3.7. Data Processing

Drug permeation across the corneal tissues (μg/cm^2^) was plotted as a function of time (min). The steady state flux J_ss_ (μg/cm^2^/h), which represents the rate of drug penetration at steady state over a given tissue area, was calculated from the slope of the regression line and the apparent permeability coefficient (P_app_) was calculated based on the following equation:

P_app_ = J_ss_/C_d_ (1)

where C_d_ (μg/mL) is the initial drug concentration in the donor solution.

### 3.8. HPLC Analysis 

Timolol maleate and brimonidine tartrate were analyzed by a HPLC system (Shimadzu, Kyoto, Japan) consisted of two LC-20AD pumps, an autosampler (SIL-20C HT), a thermostated column compartment (CTO-20AC) and a UV-Vis detector model (SPD-M20A). Separations were carried out using a C18 analytical column (150 × 4.6 mm, 3 μm, Discovery, Supelco, Bellefonte, PA, USA). The mobile phase A and B consisted of aqueous phosphate buffer (10 mΜ, pH 3 adjusted with concentrated H_3_PO_4_) and acetonitrile, respectively. The mobile phase A was filtered through 0.45 mm nylon membrane filters and degassed prior to use. The LC gradient elution program [time (min)/% mobile phase B] was set to 0/10, 3/10, 10/40, 12/10, 25/stop. The mobile phase was pumped at a flow rate of 1 mL/min, while column temperature was kept at 30 °C. The injection volume was 20 μL in all cases. The retention time was 3.9 min (λ = 245 nm) and 8.8 min (λ =300 nm) for brimonidine tartrate and timolol maleate, respectively. Calibration curves of both drugs were linear in the examined concentration ranges of 0.1–1 and 2.5–50 μg/mL with r^2^ > 0.9993 in all cases. 

### 3.9. Corneal Tissue Characterization

Immediately after completion of the permeability experiments, the tissues were removed from the Franz cells, thoroughly washed with distilled water, blotted on a filter paper to remove excess water and further characterized to evaluate the effect of the peptide hydrogel on tissue integrity. 

#### 3.9.1. Fourier Transform Infrared (FTIR) Spectroscopy

The FTIR spectra of the corneas interacting with the TM and BR formulations in PBS and in the peptide hydrogel were recorded using a Shimandzu IR-Prestige-21-FTIR spectrometer (Specac Ltd. Slough, UK) over the range of 600–4000 cm^−1^ at a resolution of 4 cm^−1^ after 54 scans for each sample. Spectra were analyzed using the program IR-Solutions (Shimadzu Co., Tokyo, Japan). Tissues were placed on the surface of the ATR crystal with the epithelial side of the tissue in contact with the crystal. 

#### 3.9.2. Differential Scanning Calorimetry (DSC) Analysis

Corneas were also characterized with differential scanning calorimetry (DSC, 204 F1 Phoenix, Netzsch, Germany). The active surface (0.19 cm^2^) of the cornea was carefully isolated from the tissue using surgical scissor. The sample was placed in aluminum pan with a perforated lid and heated from 20 °C to 80 °C at 5 °C/min at a 50 mL/min nitrogen gas flow rate.

#### 3.9.3. Hydration Content

Control and treated corneas were weighted and dried in an oven at 80 °C. After 24 h, samples were weighted again. The hydration content was calculated based on the following equation.

Hydration content (%) = (W_initial_ − W_final_) / W_initial_ (2)

where W_initial_ is the weight of corneas at the end of the ex vivo studies and W_final_ is the weight of the corneas after drying for 24 h.

### 3.10. Histological Studies

Control and treated corneas were fixed in 10% formalin. Samples were cut in 5 μm thick sections, embedded in paraffin, stained with hematoxylin and eosin and studied under an Olympus CX31 optical Microscope. Images were collected using the OLYMPUS analySIS getIT software. The same process was followed for the control sample.

### 3.11. Statistical Analysis

Data are presented as mean values ± S.D. The data were analyzed using *t*-test and significance level was set at 0.05.

## 4. Conclusions

Formulation interventions in topical ocular drug delivery, in terms of increasing drug retention and permeability and reducing frequency of administration, are essential in improving the therapeutic index in anti-glaucoma therapy. Based on the promising results of our previous study [11], in which the efficacy of an in-situ gel forming self-assembling peptide as drug carrier of timolol maleate was investigated and supported by in vitro characterization, ex vivo permeability and in vivo pharmacokinetic and pharmacodynamic studies, we intended to further enhance its therapeutic potency. In that context, the ac-(RADA)_4_-CONH_2_ self-assembling peptide hydrogel was evaluated as carrier for the topical ocular co-delivery of timolol maleate and brimonidine tartrate. The peptide hydrogel significantly enhanced the ex vivo corneal permeability of both drugs compared to the drugs’ solution, rendering it a promising co-delivery system in the treatment of glaucoma. Further in vivo pharmacokinetic and pharmacodynamic studies will be required to validate the findings of the current study.

## Figures and Tables

**Table 1:** Rheological parameters of the peptide hydrogel prior and post drug loading (0.5% strain, 1 Hz, 37 °C).

Formulation	Gelation Onset (min)	G’_max_ (kPa)	tan δ
Peptide hydrogel	39	22.6	0.05
BR + TM peptide hydrogel	30	7.1	0.28
BR peptide hydrogel	18	25.3	0.13
TM peptide hydrogel	35	8.7	0.31

**Table 2:** Apparent permeability and steady state flux values of TM and BR across porcine corneas. Data are expressed as mean ± standard deviation (*n* ≥ 4).

Formulation	J_ss_ (μg/cm^2^/min)	P_app_ (·10^−6^ cm/s)
TM peptide hydrogel	0.94 ± 0.13	2.30 ± 0.30
TM solution	0.31 ± 0.02	0.75 ± 0.05
BR peptide hydrogel	0.40 ± 0.03	3.30 ± 0.30
BR solution	0.076 ± 0.007	0.63 ± 0.05

**Table 3:** Hydration content values of the corneas at the end of the ex vivo permeability study.

Formulation	Hydration Content (%)
Control	79.65 ± 3.40
Peptide hydrogel	80.15 ± 0.32
TM + BR solution	80.30 ± 0.47
TM + BR peptide hydrogel	81.50 ± 0.60

## References

[1] Tatham A.J., Sarodia U., Gatrad F., Awan A. (2013). Eye drop instillation technique in patients with glaucoma. Eye (Lond.).

[2] Willoughby C.E., Ponzin D., Ferrari S., Lobo A., Landau K., Omidi Y. (2010). Anatomy and physiology of the human eye: Effects of mucopolysaccharidoses disease on structure and function—A review. Clin. Experiment. Ophthalmol..

[3] Gote V., Sikder S., Sicotte J., Pal D. (2019). Ocular Drug Delivery: Present Innovations and Future Challenges. J. Pharmacol. Exp. Ther..

[4] Patel A., Cholkar K., Agrahari V., Mitra A.K. (2013). Ocular drug delivery systems: An overview. World J. Pharmacol..

[5] Yu Z., Xu Q., Dong C., Lee S.S., Gao L., Li Y., D’Ortenzio M., Wu J. (2015). Self-assembling peptide nanofibrous hydrogel as a versatile drug delivery platform. Curr. Pharm. Des..

[6] Beniash E., Hartgerink J.D., Storrie H., Stendahl J.C., Stupp S.I. (2005). Self-assembling peptide amphiphile nanofiber matrices for cell entrapment. Acta Biomater..

[7] Anderson J.M., Kushwaha M., Tambralli A., Bellis S.L., Camata R.P., Jun H.-W. (2009). Osteogenic differentiation of human mesenchymal stem cells directed by extracellular matrix-mimicking ligands in a biomimetic self-assembled peptide amphiphile nanomatrix. Biomacromolecules.

[8] Zhang S., Lockshin C., Herbert A., Winter E., Rich A. (1992). Zuotin, a putative Z-DNA binding protein in Saccharomyces cerevisiae. EMBO J..

[9] Karavasili C., Panteris E., Vizirianakis I.S., Koutsopoulos S., Fatouros D.G. (2018). Chemotherapeutic Delivery from a Self-Assembling Peptide Nanofiber Hydrogel for the Management of Glioblastoma. Pharm. Res..

[10] Karavasili C., Andreadis D.A., Katsamenis O.L., Panteris E., Anastasiadou P., Kakazanis Z., Zoumpourlis V., Markopoulou C.K., Koutsopoulos S., Vizirianakis I.S. (2019). Synergistic Antitumor Potency of a Self-Assembling Peptide Hydrogel for the Local Co-delivery of Doxorubicin and Curcumin in the Treatment of Head and Neck Cancer. Mol. Pharm..

[11] Karavasili C., Komnenou A., Katsamenis O.L., Charalampidou G., Kofidou E., Andreadis D., Koutsopoulos S., Fatouros D.G. (2017). Self-Assembling Peptide Nanofiber Hydrogels for Controlled Ocular Delivery of Timolol Maleate. ACS Biomater. Sci. Eng..

[12] Liu J., Zhang L., Yang Z., Zhao X. (2011). Controlled release of paclitaxel from a self-assembling peptide hydrogel formed in situ and antitumor study in vitro. Int. J. Nanomedicine..

[13] Phipps M.C., Monte F., Mehta M., Kim H.K.W. (2016). Intraosseous Delivery of Bone Morphogenic Protein-2 Using a Self-Assembling Peptide Hydrogel. Biomacromolecules.

[14] Zhou A., Chen S., He B., Zhao W., Chen X., Jiang D. (2016). Controlled release of TGF-beta 1 from RADA self-assembling peptide hydrogel scaffolds. Drug Des. Devel. Ther..

[15] Guo H., Cui G., Yang J., Wang C., Zhu J., Zhang L., Jiang J., Shao S. (2012). Sustained delivery of VEGF from designer self-assembling peptides improves cardiac function after myocardial infarction. Biochem. Biophys. Res. Commun..

[16] Weinreb R.N., Aung T., Medeiros F.A. (2014). The pathophysiology and treatment of glaucoma: A review. JAMA.

[17] Yadav K.S., Rajpurohit R., Sharma S. (2019). Glaucoma: Current treatment and impact of advanced drug delivery systems. Life Sci..

[18] Spaeth G.L., Bernstein P., Caprioli J., Schiffman R.M. (2011). Control of intraocular pressure and fluctuation with fixed-combination brimonidine-timolol versus brimonidine or timolol monotherapy. Am. J. Ophthalmol..

[19] Arcieri E.S., Arcieri R.S., Pereira A.C.A., Andreo E.G.V., Finotti I.G.A., Sá Filho W.F. (2007). Comparing the fixed combination brimonidine-timolol versus fixed combination dorzolamide-timolol in patients with elevated intraocular pressure. Curr. Med. Res. Opin..

[20] Samy K.E., Cao Y., Kim J., Konichi da Silva N.R., Phone A., Bloomer M.M., Bhisitkul R.B., Desai T.A. (2019). Co-Delivery of Timolol and Brimonidine with a Polymer Thin-Film Intraocular Device. J. Ocul. Pharmacol. Ther. Off..

[21] Dubey A., Prabhu P. (2014). Formulation and evaluation of stimuli-sensitive hydrogels of timolol maleate and brimonidine tartrate for the treatment of glaucoma. Int. J. Pharm. Investig..

[22] Yang H., Tyagi P., Kadam R.S., Holden C.A., Kompella U.B. (2012). Hybrid Dendrimer Hydrogel/PLGA Nanoparticle Platform Sustains Drug Delivery for One Week and Antiglaucoma Effects for Four Days Following One-Time Topical Administration. ACS Nano.

[23] Wang T., Zhong X., Wang S., Lv F., Zhao X. (2012). Molecular mechanisms of RADA16-1 peptide on fast stop bleeding in rat models. Int. J. Mol. Sci..

[24] Arnold J.J., Hansen M.S., Gorman G.S., Inoue T., Rao V., Spellen S., Hunsinger R.N., Chapleau C.A., Pozzo-Miller L., Daniel Stamer W. (2013). The effect of Rho-associated kinase inhibition on the ocular penetration of timolol maleate. Investig. Ophthalmol. Vis. Sci..

[25] Zhang L., Aksan A. (2010). Fourier Transform Infrared Analysis of the Thermal Modification of Human Cornea Tissue during Conductive Keratoplasty. Appl. Spectrosc..

[26] Kampmeier J., Radt B., Birngruber R., Brinkmann R. (2000). Thermal and Biomechanical Parameters of Porcine Cornea. Cornea.

[27] Miles C.A., Avery N.C., Rodin V.V., Bailey A.J. (2005). The Increase in Denaturation Temperature Following Cross-linking of Collagen is Caused by Dehydration of the Fibres. J. Mol. Biol..

[28] Lin H.H., Ko S.M., Hsu L.R., Tsai Y.H. (1996). The preparation of norfloxacin-loaded liposomes and their in-vitro evaluation in pig’s eye. J. Pharm. Pharmacol..

[29] Sionkowska A. (2005). Thermal stability of UV-irradiated collagen in bovine lens capsules and in bovine cornea. J. Photochem. Photobiol. B Biol..

[30] Pescina S., Govoni P., Potenza A., Padula C., Santi P., Nicoli S. (2015). Development of a Convenient ex vivo Model for the Study of the Transcorneal Permeation of Drugs: Histological and Permeability Evaluation. J. Pharm. Sci..

[31] Monti D., Chetoni P., Burgalassi S., Najarro M., Saettone M. (2002). Increased corneal hydration induced by potential ocular penetration enhancers: Assessment by differential scanning calorimetry (DSC) and by desiccation. Int. J. Pharm..

[32] Stewart W.C., Stewart J.A., Holmes K.T., Leech J.N. (2000). Differences in ocular surface irritation between timolol hemihydrate and timolol maleate. Am. J. Ophthalmol..

